# Cascade of care among people with hepatitis B in New South Wales, Australia

**DOI:** 10.1111/jvh.13881

**Published:** 2023-08-08

**Authors:** Syed Hassan Bin Usman Shah, Heather Valerio, Behzad Hajarizadeh, Gail Matthews, Maryam Alavi, Gregory J. Dore

**Affiliations:** ^1^ Viral Hepatitis Clinical Research Program (VHCRP) The Kirby Institute, UNSW Sydney Australia

**Keywords:** care cascade, data linkage, HBV DNA testing, hepatitis B virus, treatment uptake

## Abstract

Hepatitis B virus (HBV) care cascade characterisation is important for monitoring HBV elimination progress. This study evaluated care cascade and factors associated with HBV DNA testing and treatment in New South Wales, Australia. HBV care cascade were determined through linkage of HBV notifications (1993–2017) to Medicare and pharmaceutical benefits schemes (2010–2018). Timely HBV DNA testing was within 4 weeks of HBV notification. Multivariate Cox proportional hazards regression evaluated factors associated with HBV DNA testing and treatment. Among 15,202 people with HBV notification, 10,479 (69%) were tested for HBV DNA. A total of 3179 (21%) initiated HBV treatment. HBV DNA testing was more likely among age ≥45 years (adjusted hazard ratio [aHR] 1.07, 95% CI: 1.02, 1.12), hepatocellular carcinoma (HCC) (aHR 1.23, 95% CI: 1.01, 1.50), coinfection (aHR 1.61, 95% CI: 1.23, 2.09), later notification (2014–2017) (aHR 1.21, 95% CI: 1.16, 1.26) and less likely among females (aHR 0.95, 95% CI: 0.91, 0.99), history of alcohol use disorder (AUD) (aHR 0.77, 95% CI: 0.66, 0.89), HCV coinfection (aHR .62, 95% CI: 0.55, 0.70) and Indigenous peoples (aHR 0.84, 95% CI: 0.71, 0.98). HBV treatment was associated with age ≥45 years (aHR 1.35, 95% CI: 1.24, 1.48), decompensated cirrhosis (aHR 2.07, 95% CI: 1.62, 2.65), HCC (aHR 2.96, 95% CI: 2.35, 3.74), HIV coinfection (aHR 4.27, 95% CI: 3.43, 5.31) and later notification (2014–2017) (aHR 1.37, 95% CI: 1.26, 1.47). HBV treatment was less likely among females (aHR 0.68, 95% CI: 0.63, 0.73) and Indigenous peoples (aHR 0.58, 95% CI: 0.42, 0.80). HBV DNA testing and treatment coverage have increased, but remain sub‐optimal among some key populations.

**Abbreviations**
APDC: admitted patient data collection
aHR: adjusted hazard ratio
AUD: alcohol use disorder
CHB: chronic hepatitis B
CheReL: centre for health record linkage
CI: confidence interval
DC: decompensated cirrhosis
GPs: general practitioners
HBV: hepatitis B virus
HCV: hepatitis C virus
HCC: hepatocellular carcinoma
HIV: human immunodeficiency virus
IQR: interquartile range
MBS: medicare benefits schedule
NHR: national hiv registry
NSW: new south wales
NCIMS: notifiable conditions information management system
PBS: pharmaceutical benefits scheme
RBDM: registry of births, deaths and marriages
WHO: world health organization's

## 1 INTRODUCTION

Chronic hepatitis B (CHB) is a global health threat, affecting more than 316 million (95% uncertainty interval 284–351) people worldwide.^1^ Hepatitis B virus (HBV)‐related morbidity and mortality continue to increase, driven by asymptomatic nature of infection prior to presenting with serious liver‐related illnesses and sub‐optimal care cascade.^2^ Less than a decade remains to achieve the World Health Organization's (WHO) goal to eliminate HBV as a public health threat. Elimination targets include reduction in mortality by 65% from 2015 levels (now proposed as an alternative population‐level HBV‐related mortality rate of ≤4/100,000 per annum^3^), an increase in diagnosis to 90% and treating 80% of those eligible for antiviral treatment.^4^, ^5^ This necessitates increased linkage to care and testing and treatment uptake^6^, ^7^; however, only 10% of people with HBV are diagnosed globally, and antiviral treatment uptake is as low as 5% in many countries,^8^ including some high‐income settings.^9^

Although therapy for CHB is generally not curative, evolving international treatment guidelines^10^, ^11^, ^12^, ^13^ and advent of well‐tolerated and highly effective nucleos(t)ide analogues drugs^10^, ^14^ have the potential for a major impact on progressive liver disease and survival.^15^ For these drugs to have the anticipated impact at the population‐level, there is a need for increased HBV screening, linkage to care, access to treatment and enhanced monitoring and evaluation of the impact of HBV programmes.^6^, ^16^ In Australia, in 2020, modelled trends demonstrated an estimated 27% of people with CHB remained undiagnosed, and a large proportion of those diagnosed were not linked to care.^9^ Monitoring and identifying the care cascade is needed to evaluate the effectiveness of existing public health programmes and provide a future framework to guide services and efforts to address HBV as a health priority.^17^ WHO has described a way of conceptualising these gaps through a ‘cascade’ or ‘care continuum’ analysis, that covers the sequential stages of hepatitis B care from diagnosis to treatment (viral suppression).^18^ If Australia is to successfully eliminate hepatitis B by 2030, it will need to improve the CHB cascade of care.^19^

New South Wales (NSW), Australia, is one of the few settings with well‐established, population‐based linked databases for all HBV notifications and prescribing and testing data.^19^, ^20^ This study aims to provide a benchmark for monitoring the progress of HBV care by developing a detailed understanding of the ‘cascade’ or ‘care continuum’ and identifying gaps in engagement in a spectrum of clinical care from diagnosis to treatment uptake among people with an HBV notification in NSW, 1993–2017.

## 2 METHODOLOGY

### 2.1 Study setting, data sources and record linkages

The data used in this study included HBV notifications linked to the data of HBV DNA testing, HBV treatment, hospital admissions, HIV notifications and mortality. Under the Public Health Act 1991, notification for all new HBV and HCV cases was made mandatory to the NSW Department of Health.^21^ These records are held in the NSW Notifiable Conditions Information Management System (NCIMS) since 1993. Hospital (public and private) admissions records were obtained through the Admitted Patient Data Collection (APDC) database, holding inpatient hospitalisation discharge records occurring from July 2001. People with HIV coinfection were identified through the National HIV Registry (NHR), holding HIV notifications since 1985. Mortality records were obtained from the Registry of Births, Deaths and Marriages (RBDM), which holds data of all deaths registered in NSW since 1993.^22^ Medicare Benefits Schedule (MBS) and Pharmaceutical Benefits Scheme (PBS) were used to obtain HBV DNA testing and HBV treatment information, respectively, since 2010.

The linkage process occurred over multiple stages. First, using demographic details (including full name, gender, date of birth and address), deterministic and probabilistic linkage of records between the notifications, hospitalisation and mortality data sets was undertaken by the NSW Centre for Health Record Linkage (CheReL).^22^ After which, CHeReL deterministically linked notifications with HIV registry records, using 2×2 name codes. The Australian Institute of Health and Welfare probabilistically linked notifications with HBV testing and treatment records using the Medicare (universal healthcare) number.^23^

### 2.2 Study population

The study population was people with HBV notification in NSW, Australia, from 1993 to 2017.

### 2.3 Study period

Linked data was extracted for the following periods: NCIMS (1993–2017), APDC (July 2001–30 June 2018); NHR (1985–2017); RBDM (1993–2018); and MBS and PBS (April 2010–2018). Administrative data sources were extracted for different durations of time, depending on available periods for linkage. Hence, the study time period was restricted (2010–2018) to optimise data coverage.

### 2.4 Exclusion criteria

Data on HBV DNA testing and treatment initiation were available since 2010; hence, HBV notifications prior to 2010 were excluded. Records were removed if death occurred within 6 months of HBV notification to allow time for HBV DNA testing and treatment initiation. Deaths prior to 2010 and post‐mortem HBV notifications were excluded. HBV notifications with no Medicare number were excluded. Further, duplicate HBV notifications were excluded from all analyses.

### 2.5 Study outcomes

The primary study outcomes were ever receiving an HBV DNA test and ever receiving HBV treatment.

Ever HBV DNA testing was defined as HBV DNA testing recorded in the MBS dataset and evaluated among all people with HBV notification. Timely HBV DNA testing was defined by an HBV DNA test occurring within 4 weeks of HBV notification. HBV DNA testing can be ordered at all healthcare service levels, including tertiary‐level hospitals, drug and alcohol services and primary care settings. The only restriction is frequency of testing, which is up to four times per year for those on antiviral therapy and annual for those not on treatment. The MBS item codes used to identify HBV DNA testing are given in Table S1.

HBV treatment initiation was defined as at least one HBV treatment prescription dispensed, recorded in the PBS data set and evaluated among all people with HBV notification. According to the latest national guidelines,^24^ antiviral therapy can be administered to those with elevated ALT and HBV DNA levels (above 2000 IU/mL [HBeAg negative] or 20,000 IU/mL [HBeAg positive]). Individuals with a history of cirrhosis require only detectable HBV DNA. HBV medicine names and their corresponding PBS item codes are given in Table S1.

Those who had a record of HBV treatment initiation and had no record of HBV DNA testing were assumed to be HBV DNA tested, given that HBV DNA testing is a prerequisite to HBV treatment (*n* = 111). However, these individuals were not included in the analysis of time from HBV notification to HBV DNA testing.

### 2.6 Exposure variables

The variable of interest was year of HBV notification, categorised by notification period (2010–2017). Age at HBV notification (≤29, 30–44 and ≥45 years), sex (male, female and missing), region of residence at the time of HBV notification (metropolitan, outer‐metropolitan, regional/rural and missing) and coinfection status (HBV only, HBV/HCV and HBV/HIV) (Table S2). Due to a small number of records with HBV/HCV/HIV coinfection (<0.5% of all included notifications), these records were merged with the HBV/HIV coinfection.^25^

History of alcohol‐use‐disorder (AUD) (no, yes) was defined as any AUD‐related hospitalisation occurring before HBV notification.^26^ Histories of decompensated cirrhosis (DC) (no, yes) and hepatocellular carcinoma (HCC) (no, yes) were defined as any DC or HCC‐related hospitalisation occurring before HBV notification (Table S3).

Aboriginal and Torres Strait Islander peoples (no, yes and missing), and country of birth (Australia, Americas, Europe, New Zealand, Africa, East Asia, Oceania, South East Asia and Western Asia, missing) was determined by an algorithm developed by the NSW Ministry of Health that pools data across multiple data sets.^27^

Among those with a record of HBV treatment, prescriber type (considered at the time of the first prescription) was identified. Prescriber type was categorised by^1^: general practitioners (GPs) (due to small numbers this includes nurse practitioners),^2^ gastroenterologists and hepatologists,^3^ infectious disease specialists and^4^ other specialists.

### 2.7 Statistical analysis

Descriptive statistics were used to report the distribution of characteristics of the cohort of all people with HBV notification meeting our inclusion criteria.

The number and proportion of individuals who have ever had a^1^ HBV DNA test,^2^ timely HBV DNA test and^3^ record of HBV treatment was calculated among all people with HBV notification occurring between 2010 and 2017. Evaluation of HBV care cascade among HBV notifications included the proportion of people who received HBV DNA testing and initiated treatment between 2010 and 2018. An estimation of prescriber type was computed overall (between 2010 and 2018) and by treatment year, that is, among chronic HBV people who received treatment within the respective analysis period (2010–2013 and 2014–2018).

Time to HBV DNA testing and treatment initiation was evaluated using time‐to‐event analysis. The observation time for HBV DNA testing and treatment began on the date of HBV notification and ended on the first recorded HBV DNA testing or on the first record of HBV treatment initiation, date of death or the end of follow‐up (31 December 2018), whichever occurred first. For time to HBV DNA testing analyses, records were dropped where the occurrence of HBV DNA testing was imputed from treatment record (*n* = 111) given no available date of HBV DNA test. For those who had a notification after testing or treatment, we assumed all these individuals were notified before HBV DNA testing or treatment initiation by adjusting the notification date 1 day prior to the first date of HBV DNA testing or treatment initiation. Kaplan–Meier cumulative hazard curves were used to estimate the cumulative probability of HBV DNA testing and treatment initiation over the study period.

Unadjusted and adjusted Cox regression analysis was used to evaluate the factors associated with HBV DNA testing and treatment initiation. For each outcome, the covariates included age at HBV notification (≤29, 30–44 and ≥45 years), sex, country of birth, ethnicity, history of AUD, DC or HCC, HCV or HIV coinfection, local health district of residence at the time of notification and year of HBV notification. The assumption of proportionality was assessed for each exposure. All variables were considered for the adjusted analysis, given significance (*p* < .02) at the unadjusted level or known or suspected clinical significance.

All analyses were performed in STATA version 16.0 (College Station).

### 2.8 Ethical approval

This publication involved information already collected by population‐based health administration registries; therefore, people have not been ‘recruited’ for the purposes of this research. Ethics approvals for the study were granted by the New South Wales Population & Health Services Research Ethics Committee, Cancer Institute New South Wales (reference number HREC/13/CIPHS/63), the Australian Institute of Health and Welfare (reference number EO2014/3/114) and the Aboriginal Health and Medical Research Council of New South Wales (reference number 1215/6).

## 3 RESULTS

### 3.1 Study participants

Between 1993 and 2017, there were 68,755 persons with an HBV notification in NSW. Among these, 15,202 people were notified during 2010–2017 and included in this analysis. Notifications excluded due to various reasons are provided in Figure S1.

Overall, 55% were male, the median age was 37 years (interquartile range [IQR] 29–50 years), 13% were born in Australia, 3% identified as Aboriginal and/or Torres Strait Islander, 3% had a history of AUD, 5% had HCV coinfection and 1% had HIV coinfection. There were 185 (1%) with a DC and 142 (1%) with an HCC diagnosis (Table 1).

**TABLE 1:** Demographic and liver disease characteristics of people with an HBV notification in NSW (2010–2017), overall and among those tested for HBV DNA and those initiating HBV treatment, *n* = 15,202

Characteristics	All HBV notified	HBV DNA tested	HBV treatment initiated
	*n* (%)^a^	*n* (%)^b^	*n* (%)^b^
Total	*n* = 15,202	*n* = 10,479^d^ (69%)	*n* = 3,179 (21%)
Age at HBV notification, median (IQR)	37 (29,50)	37 (29,50)	40 (30,53)
Age at HBV notification^c^			
≤29 years	4408 (29)	3093 (70)	836 (19)
30–44 years	5752 (38)	3938 (68)	1071 (19)
≥45 years	5040 (33)	3447 (68)	1271 (25)
Sex^c^			
Male	8337 (55)	5671 (68)	1979 (24)
Female	6843 (45)	4788 (70)	1198 (18)
Place of birth^c^			
Australia	1944 (13)	1021 (53)	323 (17)
Americas, Europe, New Zealand	847 (6)	526 (62)	158 (19)
Africa	456 (3)	374 (82)	95 (21)
Oceania/East Asia	6038 (40)	4670 (77)	1546 (26)
West/South Asia	917 (6)	648 (71)	165 (18)
Aboriginal and Torres Strait Islander^c^	468 (3)	202 (43)	46 (10)
History of alcohol‐use disorder diagnosis			
Yes	509 (3)	225 (44)	79 (16)
No	14,693 (97)	10,254 (70)	3100 (21)
Local health district at the time of HBV notification^c^			
Metropolitan NSW	6525 (43)	4613 (71)	1466 (22)
Outer metropolitan NSW	6724 (44)	4,756 (71)	1374 (20)
Regional/rural NSW	1711 (11)	1028 (60)	302 (18)
History of DC diagnosis			
Yes	185 (1)	120 (65)	90 (49)
No	15,017 (99)	10,359 (69)	3,089 (21)
History of HCC diagnosis			
Yes	142 (1)	113 (80)	101 (71)
No	15,060 (99)	10,366 (69)	3078 (20)
Coinfection status			
HBV only	14,300 (94)	10,048 (70)	2960 (21)
HBV/HCV	796 (5)	335 (42)	131 (16)
HBV/HIV	106 (1)	96 (91)	88 (83)
Year of HBV notification			
2010–2013	8700 (57)	6185 (71)	1985 (23)
2014–2017	6502 (43)	4294 (66)	1194 (18)

Abbreviations: DC, decompensated cirrhosis; HBV, hepatitis B virus; HCC, hepatocellular carcinoma; HCV, hepatitis C virus; HIV, human immunodeficiency virus; IQR, interquartile range; NSW, New South Wales.

^a^ Percentages were calculated as column percentages (denominator included the total HBV notifications, *n* = 15,202).

^**b**^ Percentages were calculated as row percentages (denominator included HBV notifications in each sub‐category).

^c^ Missing data not shown.

^d^ Includes those who initiated treatment and had no history of prior HBV DNA testing (*n* = 111).

### 3.2 HBV care cascade

**FIGURE 1:**
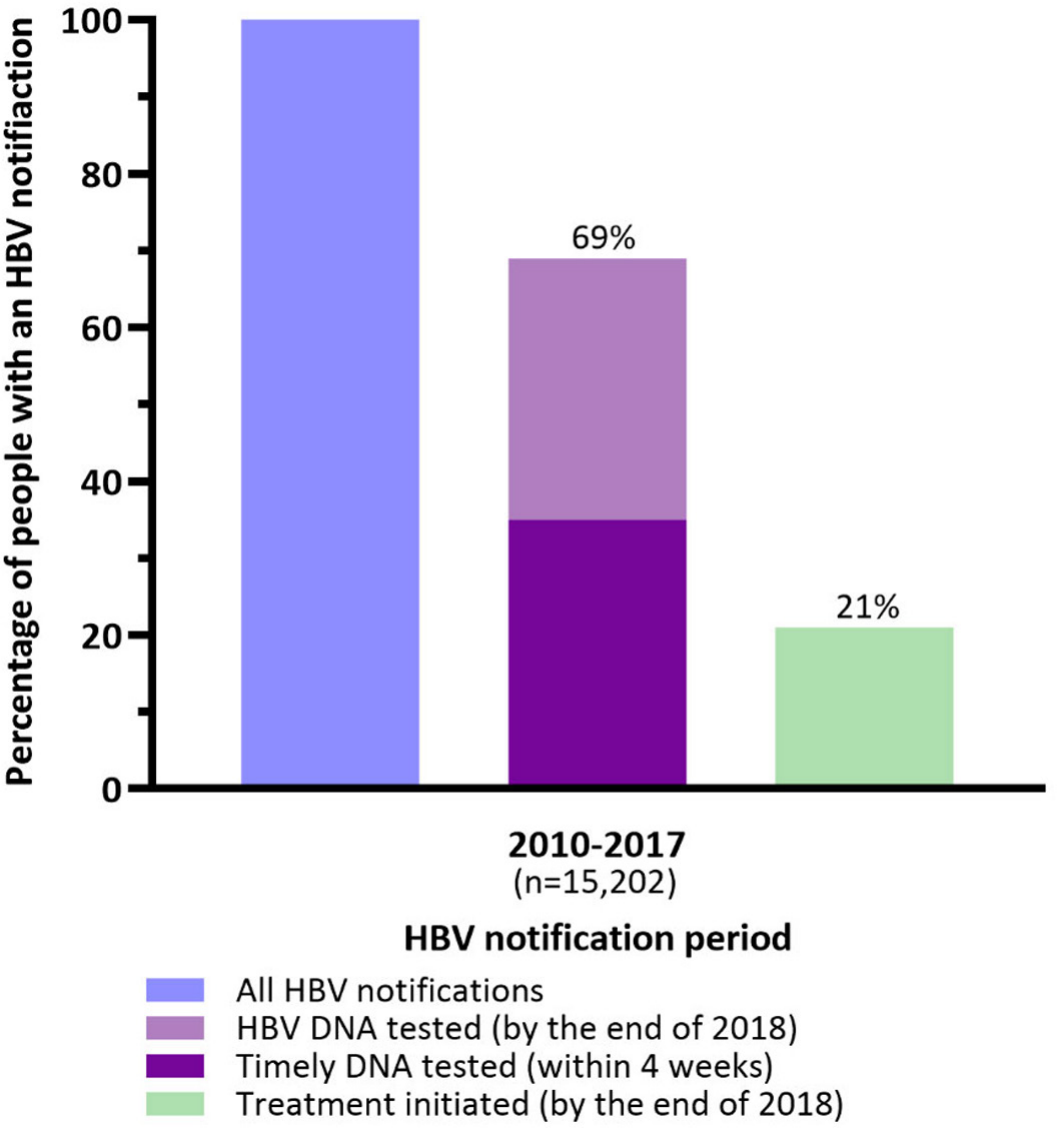
Hepatitis B virus (HBV) care cascade among people with an HBV notification (2010–2017) in New South Wales, Australia.

Of the total HBV notifications (*n* = 15,202), 10,479 (69%) were tested for HBV DNA by the end of 2018, including 5265 (35%) who received timely testing. Among those notified, 3179 (21%) received treatment (Figure 1). Of those tested for HBV DNA, 3179/10,479 (30%) were treated.

The proportion of HBV DNA testing uptake was higher in younger females (29 years and below) than those aged 45 years and above (74% vs. 68%). In contrast, young males had slightly lower HBV DNA testing rates than older men (66% vs. 69%) (Table S4).

### 3.3 HBV DNA testing

Among all individuals tested for HBV DNA (*n* = 10,479), 5265 (50%) had timely DNA testing. Compared to testing after 4 weeks (delayed), the probability of timely testing was higher among older people (median age 35 years vs. 38 years), those living in the metropolitan NSW (33% vs. 36%), those who had a history of HCC (37% vs. 40%) and those notified in the later period (26% vs. 39%). Further, the probability of timely DNA testing was lower among Aboriginal and Torres Strait Islander people (26% vs. 16%), those with a history of AUD (25% vs. 17%), DC (36% vs. 24%) and those notified between 2010 and 2013 (39% vs. 31%) (Table S5).

**FIGURE 2:**
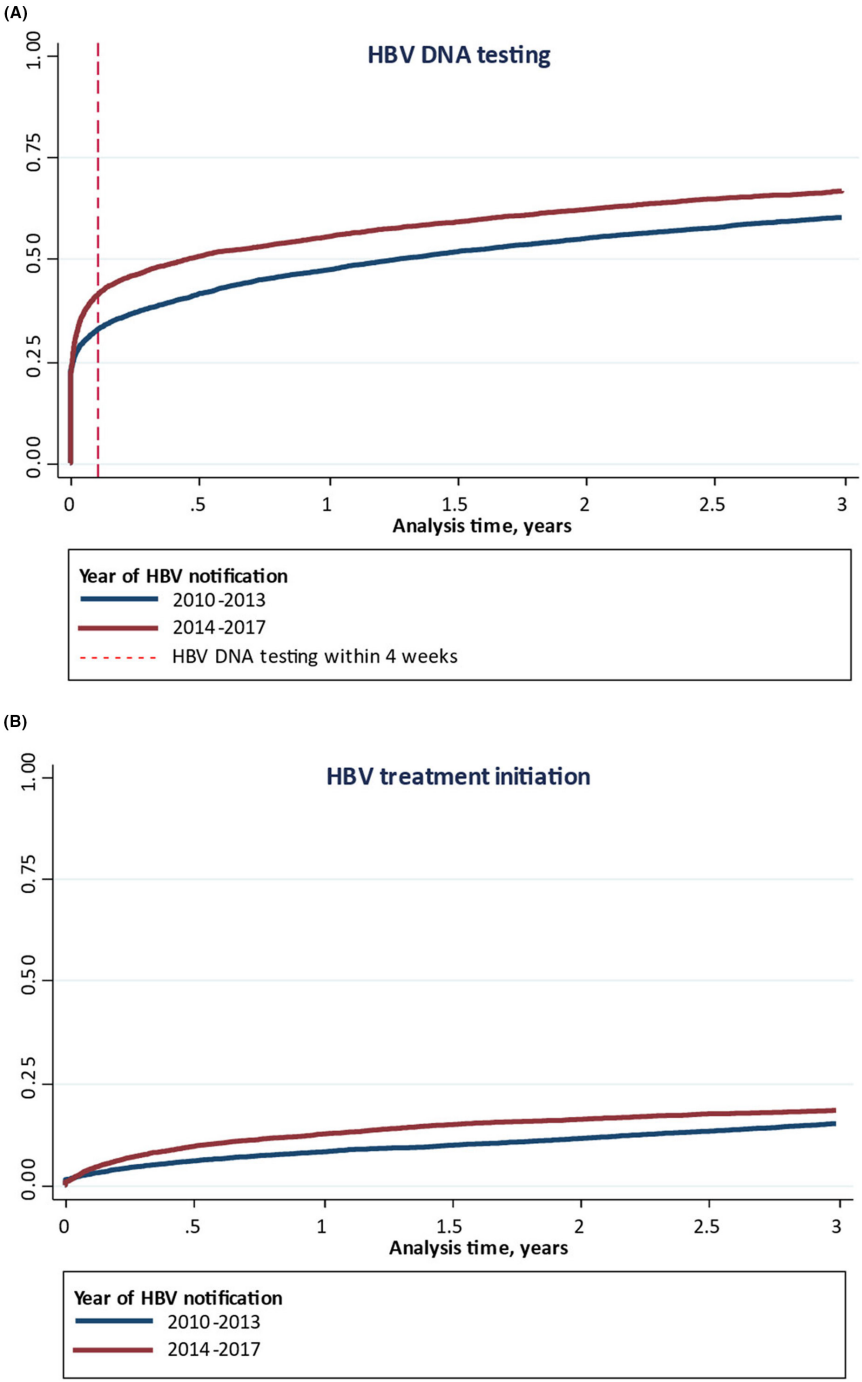
Cumulative probability of (A) hepatitis B virus (HBV) DNA testing and (B) HBV treatment initiation over 3 years among people with an HBV notification in New South Wales, by time period of HBV notification (2010–2013 versus 2014–2017).

The cumulative probability of HBV DNA testing over 3 years was higher among people notified during 2014–2017 (66.6%; 95% confidence interval [CI]: 65.3, 67.8) compared to those notified during 2010–2013 (60.5%; 95% CI: 59.5, 61.5; Figure 2).

At 28 days following HBV notification (timely testing), the cumulative probability of HBV DNA testing was higher among those who were notified during 2014–2017 (39.5%; 95% CI: 38.3, 40.7) compared to those notified during 2010–2013 (31.4%; 95% CI: 30.4, 32.4) (Figure 2).

### 3.4 HBV treatment initiation

HBV treatment was initiated in 3179 people (21%). The median age of individuals treated was 40 years (IQR: 30–53 years). The probability of treatment initiation was higher among older people 45 years or above (25%) compared to 30–44 years (19%) and ≤ 29 years (19%). Aboriginal and/or Torres Strait Islander people (10%), females (18%), and individuals with a history of AUD (16%), HCV coinfection (16%) had low treatment uptake. In contrast, treatment initiation was considerably high among individuals with a history of HCC, DC and HIV coinfection (71%, 49% and 83% respectively) (Table S6).

**FIGURE 3:**
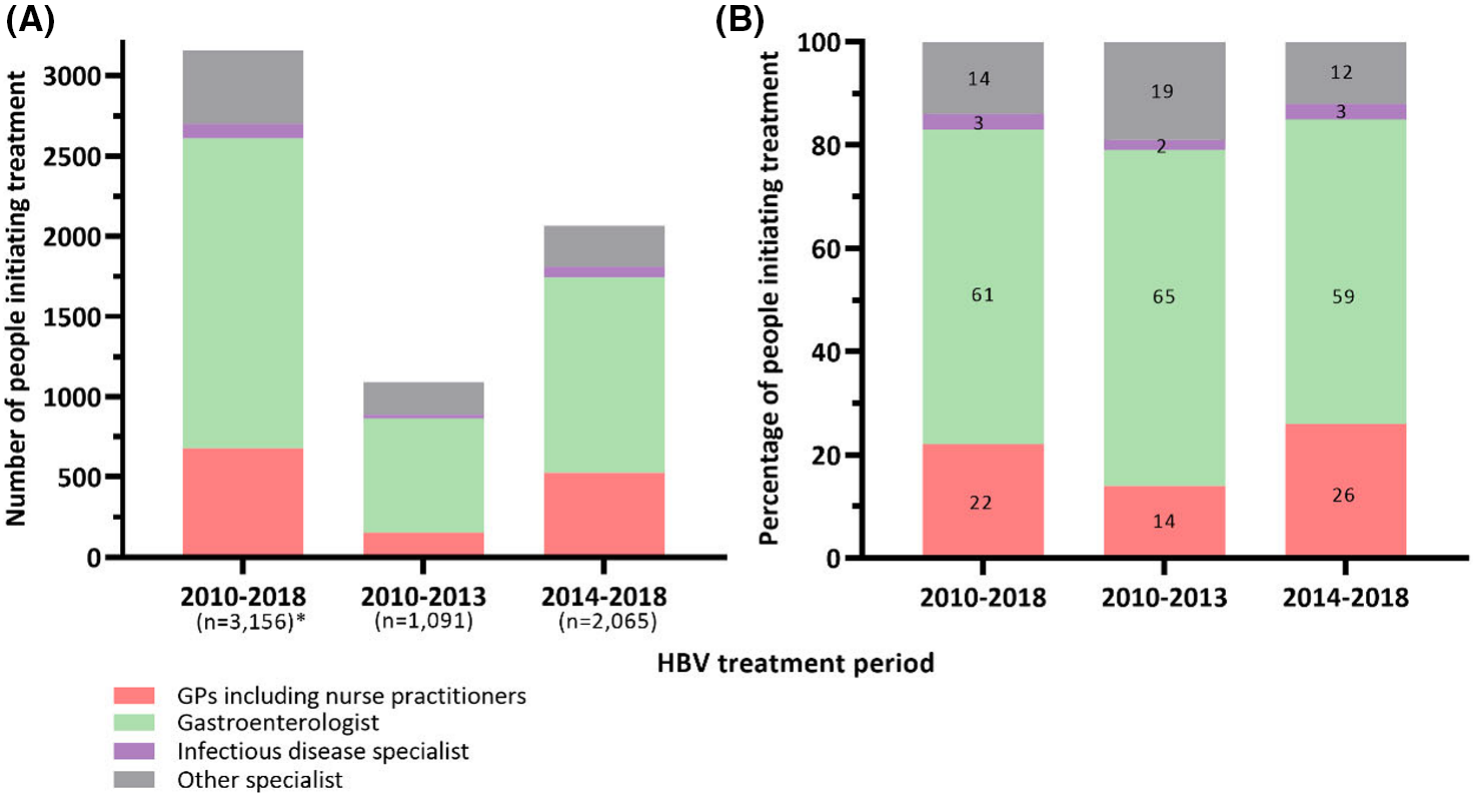
The number (A) and proportion (B) of hepatitis B virus (HBV) treatment initiations by prescriber type and treatment year in New South Wales, Australia.

Among those treated (*n* = 3,179), the proportion of individuals initiated on treatment by a gastroenterologist declined from 65% (713 of 1091) in 2010–2013 to 59% (1217 of 2065) in 2014–2018. In contrast, the proportion of GP‐prescribed HBV treatment initiation increased from 14% (153 of 1091) in 2010–2013 to 26% (526 of 2065) in 2014–2018 (Figure 3).

The cumulative probability of HBV treatment initiation over 3 years was higher among people notified during 2014–2017 (18.1%; 95% CI: 17.2, 19.2) compared to those notified during 2010–2013 (14.8%; 95% CI: 14.0, 15.5; Figure 2).

### 3.5 Factors associated with HBV DNA testing

In adjusted analysis, the rate of HBV DNA testing was higher among people aged ≥45 years compared to those ≤29 years (aHR 1.07, 95% CI: 1.02, 1.12). HBV DNA testing was more likely among those: born overseas (Africa; aHR 1.61, 95% CI: 1.42, 1.82, Oceania/ East Asia; aHR 1.50, 95% CI: 1.39, 1.63, West/ South Asia; aHR 1.31, 95% CI: 1.18, 1.46), with HCC (aHR 1.23, 95% CI: 1.01, 1.50), HIV coinfection (aHR 1.61, 95% CI: 1.23, 2.09) and later period (2014–2017) of HBV notification (aHR 1.21, 95% CI: 1.16, 1.26). HBV DNA testing was less likely among females (aHR 0.95, 95% CI: 0.91, 0.99), and among those: with a history of alcohol use disorder (aHR 0.77, 95% CI: 0.66, 0.89), with a history of HCV coinfection (aHR 0.62, 95% CI: 0.55, 0.70), HBV notified in regional/rural NSW regions (aHR 0.88, 95% CI: 0.81, 0.94) and Aboriginal and Torres Strait Islander people (aHR 0.84, 95% CI: 0.71, 0.98) (Table 2).

**TABLE 2:** Cox proportional hazards regression analysis (unadjusted and adjusted), evaluating factors associated with ever HBV DNA testing among people with an HBV notification in NSW (2010–2017), *n* = 10,479

Characteristics	HBV tested *n* = 10,479 (%)	Unadjusted HR (95% CI)	*p*‐value	Adjusted HR (95% CI)	*p*‐value
Age at HBV notification					
≤29 years	3093 (29)	Reference		Reference	
30–44 years	3938 (38)	0.98 (0.94, 1.03)	.650	1.02 (0.97, 1.07)	.356
≥45 years	3447 (33)	1.04 (0.99, 1.09)	.084	1.07 (1.02, 1.12)	.005
Sex^a^					
Male	5671 (54)	Reference		Reference	
Female	4788 (46)	1.02 (0.98, 1.06)	.197	0.95 (0.91, 0.99)	.023
Place of birth^a^					
Australia	1021 (10)	Reference		Reference	
Americas, Europe, New Zealand	526 (5)	1.30 (1.17, 1.45)	<.001	1.03 (0.92, 1.16)	.529
Africa	374 (4)	1.95 (1.73, 2.20)	<.001	1.61 (1.42, 1.82)	<.001
Oceania/East Asia	4,670 (45)	1.88 (1.76, 2.02)	<.001	1.50 (1.39, 1.63)	<.001
West/South Asia	648 (6)	1.68 (1.52, 1.86)	<.001	1.31 (1.18, 1.46)	<.001
Aboriginal and Torres Strait Islander^a^					
No	6888 (66)	Reference		Reference	
Yes	202 (2)	0.43 (0.37, 0.50)	<.001	0.84 (0.71, 0.98)	.037
History of alcohol‐use disorder diagnosis					
No	10,254 (98)	Reference		Reference	
Yes	225 (2)	0.47 (0.41, 0.54)	<.001	0.77 (0.66, 0.89)	.001
Local Health District at the time of HBV notification^a^					
Metropolitan NSW	4613 (44)	Reference		Reference	
Outer metropolitan NSW	4756 (45)	1.00 (0.96, 1.04)	.760	1.02 (0.98, 1.06)	.234
Regional/rural NSW	1028 (10)	0.75 (0.70, 0.80)	<.001	0.88 (0.81, 0.94)	.001
History of DC diagnosis					
No	10,359 (99)	Reference		Reference	
Yes	120 (1)	0.87 (0.72, 1.05)	.156	0.98 (0.80, 1.20)	.872
History of HCC diagnosis					
No	10,366 (99)	Reference		Reference	
Yes	113 (1)	1.31 (1.09, 1.58)	.004	1.23 (1.01, 1.50)	.037
Coinfection status					
HBV only	10,048 (96)	Reference		Reference	
HBV/HCV	335 (3)	0.45 (0.40, 0.50)	<.001	0.62 (0.55, 0.70)	<.001
HBV/HIV	96 (1)	1.47 (1.13, 1.91)	.003	1.61 (1.23, 2.09)	<.001
Year of HBV notification					
2010–2013	6185 (59)	Reference		Reference	
2014–2017	4294 (41)	1.18 (1.13, 1.23)	<.001	1.21 (1.16, 1.26)	<.001

Abbreviations: DC, decompensated cirrhosis; HBV, hepatitis B virus; HCC, hepatocellular carcinoma; HCV, hepatitis C virus; HIV, human immunodeficiency virus; IQR, interquartile range; NSW, New South Wales.

^a^ Missing data not shown.

### 3.6 Factors associated with HBV treatment initiation

In adjusted analysis, the rate of HBV treatment initiation was higher among people aged ≥45 years compared to those ≤29 years (aHR 1.35, 95% CI: 1.24, 1.48). HBV treatment initiation was more likely among those; born overseas (Oceania/ East Asia (aHR 1.51, 95% CI: 1.32, 1.73), and among those: with a history of DC (aHR 2.07, 95% CI: 1.62, 2.65), HCC (aHR 2.96, 95% CI: 2.35, 3.74), HIV coinfection (aHR 4.27, 95% CI: 3.43, 5.31) and later period (2014–2017) of HBV notification (aHR 1.37, 95% CI: 1.26, 1.47). HBV treatment initiation was less likely among females (aHR 0.68, 95% CI: 0.63, 0.73) and Aboriginal and Torres Strait Islander people (aHR 0.58, 95% CI: 0.42, 0.80) (Table 3).

**TABLE 3:** Cox proportional hazards regression analysis (unadjusted and adjusted), evaluating factors associated with HBV treatment among people with an HBV notification in NSW (2010–2017), *n* = 3,179

Characteristics	HBV treated *n* = 3179 (%)	Unadjusted HR (95% CI)	*p*‐value	Adjusted HR (95% CI)	*p*‐value
Age at HBV notification					
≤29 years	836 (26)	Reference		Reference	
30–44 years	1071 (34)	1.03 (0.94, 1.13)	.487	0.98 (0.89, 1.07)	.691
≥45 years	1271 (40)	1.51 (1.39, 1.65)	<.001	1.35 (1.24, 1.48)	<.001
Sex^a^					
Male	1979 (62)	Reference		Reference	
Female	1198 (38)	0.69 (0.64, 0.74)	<.001	0.68 (0.63, 0.73)	<.001
Place of birth^a^					
Australia	323 (10)	Reference		Reference	
Americas, Europe, New Zealand	158 (5)	1.18 (0.98, 1.43)	.079	0.95 (0.77, 1.16)	.624
Africa	95 (3)	1.21 (0.96, 1.52)	.094	1.20 (0.95, 1.53)	.115
Oceania/East Asia	1546 (49)	1.58 (1.40, 1.78)	<.001	1.51 (1.32, 1.73)	<.001
West/South Asia	165 (5)	1.11 (0.92, 1.34)	.265	1.02 (0.84, 1.25)	.777
Aboriginal and Torres Strait Islander^a^					
No	2214 (70)	Reference		Reference	
Yes	46 (1)	0.39 (0.29, 0.52)	<.001	0.58 (0.42, 0.80)	.001
History of alcohol‐use disorder diagnosis					
No	3100 (98)	Reference		Reference	
Yes	79 (2)	0.71 (0.56, 0.88)	.003	0.79 (0.62, 1.02)	.072
Local health district at the time of HBV notification^a^					
Metropolitan NSW	1466 (46)	Reference		Reference	
Outer metropolitan NSW	1374 (43)	0.89 (0.82, 0.96)	.002	0.92 (0.86, 1.00)	.054
Regional/rural NSW	302 (10)	0.76 (0.67, 0.86)	<.001	0.89 (0.78, 1.01)	.091
History of DC diagnosis					
No	3089 (97)	Reference		Reference	
Yes	90 (3)	3.24 (2.62, 3.99)	<.001	2.07 (1.62, 2.65)	<.001
History of HCC diagnosis					
No	3078 (97)	Reference		Reference	
Yes	101 (3)	5.67 (4.65, 6.92)	<.001	2.96 (2.35, 3.74)	<.001
Coinfection status					
HBV only	2960 (93)	Reference		Reference	
HBV/HCV	131 (4)	0.75 (0.63, 0.90)	.002	0.86 (0.71, 1.04)	.140
HBV/HIV	88 (3)	4.39 (3.55, 5.43)	<.001	4.27 (3.43, 5.31)	<.001
Year of HBV notification					
2010–2013	1985 (62)	Reference		Reference	
2014–2017	1194 (38)	1.27 (1.17, 1.37)	<.001	1.37 (1.26, 1.47)	<.001

Abbreviations: DC, decompensated cirrhosis; HBV, hepatitis B virus; HCC, hepatocellular carcinoma; HCV, hepatitis C virus; HIV, human immunodeficiency virus; IQR, interquartile range; NSW, New South Wales.

^a^ Missing data not shown.

## 4 DISCUSSION

Our study demonstrates major gaps in the care cascade for people living with CHB, despite government‐funded testing and antiviral therapy. This study focussed on two key elements of the care cascade post‐notification: HBV DNA testing and HBV treatment initiation. In Australia, HBV notification is predominantly based on serological evidence of chronic infection. Although most people with a recent HBV notification received HBV DNA testing, treatment uptake remains sub‐optimal. Encouragingly for HBV elimination progress, HBV DNA testing and treatment uptake improved over time. The findings, particularly those outlining sub‐populations with poorer care cascade outcomes, will inform public health policy and practice.

Enhanced HBV DNA testing and treatment coverage are clear priorities within the Australian hepatitis B elimination strategy.^28^ The proportion with HBV DNA testing among those with HBV notification from 2010 was 69%, with testing within 4 weeks of notification in 35%. The relatively higher levels of timely HBV DNA testing in the more recent period (39% in 2014–2017 vs. 31% in 2010–2013) suggests improving awareness of the importance of HBV DNA testing in clinical assessment. But, given all people with CHB are recommended for HBV DNA testing,^24^ there remains a clear cascade of care gap. Lower HBV DNA testing levels among those with a history of AUD, HCV coinfection, Aboriginal and Torres Strait Islander people, females and individuals living in the regional/rural regions of NSW is concerning, particularly as factors such as AUD and HCV are cofactors for liver disease progression. Some of these factors may be linked to lack of consistent engagement in care or difficulties in accessing testing. Further improvements in HBV DNA testing levels should be viewed in the context of broader and more innovative HBV screening strategies. Opportunistic testing of people at risk of HBV,^24^ including consideration of one‐off universal HBV testing and simplified and timely diagnostic platforms providing single‐visit testing through the utilisation of point‐of‐care technologies, are required.^29^

Achieving higher HBV therapeutic coverage among the eligible population is a clear goal on the pathway to HBV elimination.^24^ Among all HBV notifications 2010–2017, 21% initiated HBV treatment, leaving a clear gap in linkage to treatment.^30^ Although the treatment initiation proportion in our study among a population more recently notified was suboptimal, it was much higher than a recent national estimate (11%) for all people with CHB.^31^ The relatively higher HBV treatment initiation relates to NSW having higher coverage than other Australian jurisdictions,^9^, ^31^ and higher uptake among those more recently notified.

Although the HBV treatment uptake would appear sub‐optimal, there is uncertainty regarding the proportion of people with CHB in Australia who are eligible for treatment. Government‐funded antiviral therapy is generally provided for those with elevated ALT and HBV DNA levels above 2000 IU/mL (HBeAg negative) or 20,000 IU/mL (HBeAg positive), with the exception being those with cirrhosis who only require detectable HBV DNA.^24^ This proportion, however, will vary based on the ALT normal range, with higher eligibility if the newer upper limits of 19 (females) and 30 (males)^24^ are utilised. A recent modelling study estimates that around 30% of all people living with CHB in Australia are eligible for antiviral treatment.^32^ A national cohort, REACH‐B, has recently commenced with planned broad recruitment with a key objective to estimate this proportion which could be greater than 30%.

In our study, HBV treatment initiation was relatively high among specific subgroups, particularly related to less strict eligibility criteria. Higher treatment coverage in those with end‐stage liver disease, either DC or HCC, is expected, given broader eligibility for people with cirrhosis. Higher treatment coverage among those with HIV coinfection relates to the recommendation for all people living with HIV/HBV to be on HBV‐active antiretroviral therapy (ART). Similar to HIV/HCV coinfection,^33^ it would appear that viral hepatitis elimination efforts are advanced in those with HIV/HBV coinfection. Females, Aboriginal and Torres Strait Islander people, and those with a history of AUD were less likely to initiate treatment. Lower HBV treatment coverage among females would suggest that clinicians are not employing the lower ALT upper limit of normal (19 IU/mL) to guide treatment initiation. Lower treatment coverage among those with AUD is particularly concerning, given the higher risk of progression to end‐stage liver disease.^34^

High uptake of HCV antiviral therapy in Australia has related to unrestricted access, including involvement of non‐specialists in prescribing and diverse models of care.^35^ A similar approach in HBV care with regard to involvement of non‐specialists and diverse models of care is required to enhance treatment coverage. Models of care should encompass close monitoring, given the dynamic nature of CHB, as those currently not recommended for the treatment have the potential to become treatment eligible. Estimates suggest that over 2 years, around ~20% of people progress from treatment ineligible to eligible.^36^ To improve the HBV care cascade, the Australian government, in 2015, recommended a simplified HBV antiviral prescribing and dispensing procedure involving GPs, nurse practitioners and pharmacists.^37^ In Australia, between 2016 and 2020, the proportion of HBV treatment prescribed by GPs increased from 17% to 23%.^31^ A corresponding decrease from 76% to 67% in the proportion of specialist‐prescribed treatment was observed.^30^ Encouraging and broadening antiviral therapy prescription through GPs can enhance engagement with HBV care.

Older age was associated with higher HBV DNA testing and treatment uptake compared to less than 30 years, probably related to more active and more advanced disease, a result that a previous Canadian study has corroborated.^38^ Females were less likely to undergo HBV DNA testing and initiate treatment than males. The low HBV DNA testing results were in contrast to the HCV cascade of care studies, where females had more timely HCV RNA testing but delayed treatment initiation.^33^, ^39^ The overall testing levels were low despite a higher proportion of HBV DNA testing among younger females, presumably related to antenatal care. This stresses concerted efforts to enhance testing in older women. Issues in accessing care, inadequate information about the disease, its management and follow‐up procedures and distrust of the healthcare system at the patient level are a few potential reasons for poorer HBV care cascade among females.^40^, ^41^ This highlights the need to enhance efforts towards strategies that educate and encourage them to use these pregnancy‐safe medicines. Moreover, ensuring adequate antenatal screening and vaccination aligns with national and state‐based programmes.^28^ A priority area in creating a supportive environment for increased engagement in testing and treatment is to ensure a high level of knowledge and health literacy among females and young adults.^28^

Interestingly, HBV care cascade rates were higher among people born overseas, predominantly in people from the Asia‐Pacific region, similar to earlier estimates.^28^, ^31^ It is unclear why those born overseas had better linkage to care. Further information is required on CHB phase and ALT and HBV DNA patterns among different ethnicities and country of birth populations, as this may influence treatment eligibility.

Additional factors associated with lower HBV DNA testing and treatment uptake were indigenous Australian identification and area of residence. Geographical remoteness is a key consideration since people living in the outer or regional regions of NSW were less likely to get tested and start treatment.^28^, ^31^ Demographic distribution may play a role in the care cascade since people tend to concentrate in larger urban areas in ethnically diverse environments,^38^ have more specialist access and have high treatment uptake.^30^ Enhanced HBV‐specific clinical services are clearly required in regional and rural settings, including upskilling of GPs and nurse practitioners.

Simplified models of HBV care and strengthened responses to hepatitis B are needed to enhance the care cascade.^28^ This includes reducing the number of visits required to diagnose HBV, linkage to treatment and ongoing care and treatment settings like community drug treatment clinics,^33^ which can ensure more equitable HBV care. HBV DNA point‐of‐care testing should be evaluated, although the situation differs to HCV RNA testing due to the much broader HCV antiviral therapy eligibility and defined treatment duration.

People with a history of AUD have a higher risk of cirrhosis^42^; poor HBV DNA testing and treatment initiation observed among this group are therefore of great concern. In some settings, those with a history of AUD are socially marginalised, concerted efforts are required to engage with a population that suffers considerable stigma and discrimination.^43^

Lower HBV DNA testing and treatment uptake among people with HCV coinfection is also of concern, given accelerated progression of liver disease^44^ and recommendations to prioritise treatment.^45^ The anecdotal reports of HBV decompensation among HBV/HCV‐coinfected people receiving early generation DAAs has been proposed as an explanation for lower HCV antiviral therapy uptake in this population.^46^ However, the explanation for lower HBV treatment initiation is unclear. Most people with HCV coinfection have a history of injecting drug use, therefore social marginalisation may also be a factor. People with HCV coinfection and clinicians should receive further education, counselling and training on the current guidelines to strengthen the connection between at‐risk populations and the healthcare workforce and to ensure a high antiviral treatment uptake among those with HCV coinfection.

Enhanced HBV care cascade is crucial to reduce liver disease burden, including development of end‐stage liver disease (DC and HCC).^47^ If untreated, the 5‐year survival rate is 14%–45% after DC^48^ and 18%–20% after HCC.^49^ Although individuals with a history of DC and HCC had a higher HBV treatment initiation rate, for many, this intervention is too late to prevent HBV‐related mortality. Thus, development of end‐stage liver disease without prior antiviral therapy for several years should be regarded as a major missed opportunity for enhanced care. Monitoring of late antiviral therapy initiation should be undertaken to guide policy and practice.

## 5 LIMITATIONS

There are several limitations to our study. First, HBV notifications in NSW are predominantly based on evidence of chronic infection as defined by HBV serology. Thus, the number of individuals with active viral replication could not be evaluated. Second, the timing of notification in some cases were clearly following HBV diagnosis, as the HBV DNA testing or treatment initiation dates were prior to notification. In these cases, HBV notification date was revised to the earlier of HBV DNA testing and/or treatment initiation. Third, using administrative data to define AUD has clear limitations, given the low sensitivity of administrative data (68% sensitivity and 97% specificity for the diagnosis of heavy alcohol intake).^50^ Fourth, some HBV DNA testing is undertaken through funding streams outside Medicare, such as in public hospitals and other state‐based services, and therefore not included in the data set. Finally, since HBV therapy is a lifelong treatment, our data provided treatment initiation, but information on treatment adherence was unavailable.

## 6 CONCLUSION

In conclusion, this population‐level study provides evidence for an improved HBV care cascade during 2014–2018; strategies to engage high‐risk populations and facilitate elimination are still required. Of those tested, about half received timely HBV DNA testing, higher in the later period. However, treatment uptake among some key population groups like females, Indigenous Australian ethnicity and those with a history of AUD and HCV coinfection was alarmingly low. Innovative and culturally appropriate strategies to enhance linkage to care, including HBV screening, point‐of‐care testing and treatment initiation among eligible individuals, are necessary to engage people with ongoing risk behaviour or with mild liver disease.

## AUTHORS CONTRIBUTIONS

Syed Hassan Bin Usman Shah, Heather Valerio, Behzad Hajarizadeh and Gregory J Dore contributed to study conception and design, data acquisition and analysis, interpretation of findings, and drafting of the article; Gail Matthews, Maryam Alavi and Behzad Hajarizadeh contributed to data acquisition and analysis and interpretation of findings.

## FUNDING INFORMATION

The Kirby Institute is funded by the Australian Government Department of Health, under the agreement ID number 2‐D3X513. This publication is part of the Bloodborne viruses and sexually transmissible infections Research, Strategic Interventions and Evaluation (BRISE) program, funded by the New South Wales Ministry of Health. Gregory J Dore is supported by an NHMRC of Australia Program Grant (1150078) and Investigator Fellowship (2008276).

## CONFLICT OF INTEREST STATEMENT

Gregory J Dore has received research support from Gilead Sciences, Merck and AbbVie. Gail Matthews has received research support from Gilead Sciences and AbbVie. Other authors have no commercial relationships that might pose a conflict of interest in connection with this article.

## ETHICS STATEMENT

This publication involved information already collected by population‐based health administration registries; therefore, people have not been ‘recruited’ for the purposes of this research. Ethics approvals for the study were granted by the New South Wales Population & Health Services Research Ethics Committee, Cancer Institute New South Wales (reference number HREC/13/ CIPHS/63), the Australian Institute of Health and Welfare (reference number EO2014/3/114) and the Aboriginal Health and Medical Research Council of New South Wales (reference number 1215/6).

## Supporting information


Figure S1.

Table S1.
Table S2.
Table S3.
Table S4.
Table S5.
Table S6.

## Data Availability

This publication involved information collected by population‐based health administration registries. Data used for this research cannot be deposited on servers other than those approved by ethics committees. This publication has used highly sensitive health information by linking several administrative datasets. De‐identified linked information has been provided to the research team under strict privacy regulations. Except in the form of conclusions drawn from the data, researchers do not have permission to disclose any data to any person other than those authorised for the research project.
