# Supplementary material for: Cascade of care among people with hepatitis B in New South Wales, Australia
Source: J Viral Hepat. 2023 Aug 8;30(12):926–38. doi: 10.1111/jvh.13881 (PMC10946799; doi:10.1111/jvh.13881)
Supplement: Supplementary file 1 — Figure S1. [file JVH-30-926-s001.docx]

**Figure S1: Derivation of cohort and HBV cascade of care groups**

HBV notifications as of end 2017, n=68,755

HBV notifications for analysis, n=15,202

n=53,553 excluded:

- 51,281 notified pre-2010
- 206 died within 6 months of HBV notification
- 2 post-mortem HBV notification
- 1,340 unlinked to Medicare
- 724 identifiable duplicate records

HBV DNA test, n=10,368 (68%)

- Timely test, n=5,265 (35%)
- Delayed, n= 5,103 (33%)

Treated*

n=3,179

(21%)

Treated but no record of test

n=111 (1%)

No DNA test recorded

n=4,723 (31%)

Untreated*

n=12,023

(79%)

^*^Percentages were calculated from all HBV notifications for analysis (n=15,202)
