# Supplementary material for: Cascade of care among people with hepatitis B in New South Wales, Australia
Source: J Viral Hepat. 2023 Aug 8;30(12):926–38. doi: 10.1111/jvh.13881 (PMC10946799; doi:10.1111/jvh.13881)
Supplement: Supplementary file 2 — Table S1. Table S2. Table S3. Table S4. Table S5. Table S6. [file JVH-30-926-s002.docx]

**Table S1: Pharmaceutical Benefits Scheme (PBS) and Medicare Benefits Scheme** (**MBS) item codes used to define HBV medicines and HBV DNA testing among people with an HBV notification**

| **HBV treatment**  **(Medicine names)** | **PBS item codes** |
| --- | --- |
| Entecavir | 05711N, 05712P, 05712P, 09602J, 09603K, 10279B, 10353X |
| Tenofovir | 06358P, 09563H, 10310P, 11142K, 11155D, 11978K, 11982P, 11992E |
| Interferon alfa-2a | 05759D, 05760E, 05761F, 05762G, 05763H, 05764J, 05765K, 05766L, 05767M, 05768N, 06210W, 06211X, 06212Y, 06213B, 06218G, 06219H, 06244P, 06245Q, 06246R, 06253D, 06254E, 06255F, 10291P, 10292Q, 10316Y, 10317B, 10339E, 10340F, 10354Y, 10369R, 10370T, 10371W |
| Peginterferon alfa-2a | 06439X, 06449K, 09515T, 09516W, 10278Y, 10280C, 11037X, 11416W |
| Telbivudine | 09562G, 09630W, 10372X |
| Lamivudine | 05770Q, 05771R, 06257H, 06217C, 10315X, 10338D, 10311Q, 10320E, 10348P, 10357D, 10305J, 10345L, 10843H, 10284G |
| Adefovir Dipivoxil | 05606C, 06450L, 10290N |
| Combination medicines for HIV/HBV | 11099E, 11113X, 11114Y, 11104K, 11146P, 11306C, 10347N, 11276L, 11149T, 11296M, 12506F, 11732L, 11649D, 11955F, 12542D |
|  | **MBS item codes** |
| **HBV DNA testing** | 69482, 69483 |

**Table S2: List of exposure variables**

| **Characteristics** |
| --- |
| **Age at HBV notification** |
| ≤29 years |
| 30-44 |
| ≥45 years |
| **Sex** |
| Male |
| Female  Missing |
| **Place of birth** |
| Australia |
| Americas, Europe, New Zealand |
| Africa |
| Oceania/ East Asia |
| West/ South Asia |
| Missing |
| **Aboriginal and Torres Strait Islander** |
| Yes |
| No |
| Missing |
| **History of alcohol-use disorder diagnosis** |
| Yes |
| No |
| **Local Health District at the time of HBV notification** |
| Metropolitan NSW |
| Outer metropolitan NSW |
| Regional/rural NSW |
| Missing |
| **History of DC diagnosis** |
| Yes |
| No |
| **History of HCC diagnosis** |
| Yes |
| No |
| **Coinfection status** |
| HBV only |
| HBV/HCV |
| HBV/HIV |
| **Prescriber type** |
| General practitioners (GPs) (due to small numbers this includes nurse practitioners) |
| Gastroenterologists and hepatologists |
| Infectious disease specialists |
| Other specialists |

**Table S3: International Classification of Diseases** (**ICD-10) codes used to define alcohol use disorder and end-stage liver disease among all NSW people with an HBV notification**

| **Description** | **ICD-10 codes** |
| --- | --- |
| **Alcohol-use disorder** |  |
| alcohol-induced Pseudo-Cushing's syndrome | E24.4 |
| mental and behavioural disorders due to use of alcohol | F10 |
| degeneration of nervous system due to alcohol | G31.2 |
| alcohol polyneuropathy | G62.1 |
| alcoholic cardiomyopathy | I42.6 |
| alcohol myopathy | G72.1 |
| alcohol rehabilitation | Z50.2 |
| alcohol abuse counselling and surveillance | Z71.4 |
| **End-stage liver disease** |  |
| Decompensated cirrhosis |  |
| ascites | R18 |
| bleeding oesophageal varices | I85.0 and I98.3 |
| chronic hepatic failure (including hepatic encephalopathy) | K72.1 and K72.9 |
| alcoholic hepatic failure | K70.4 |
| hepatorenal syndrome | K76.7 |
| Hepatocellular carcinoma |  |
| hepatocellular carcinoma | C22.0 |

**Table S4: HBV DNA testing uptake by age groups and gender**

| **Age groups** | **Male n(%)** | **Female n(%)** |
| --- | --- | --- |
| ≤ 29 years | 1,405/2,114 (66) | 1,679/2,284 (74) |
| 30-44 | 2,197/3,208 (68) | 1,732/2,534 (68) |
| ≥ 45 years | 2,069/3,014 (69) | 1,376/2,024 (68) |
| **Total** | 5,671/8,336 (68) | 4,787/6,842 (70) |

**Table S5: Demographic and liver disease characteristics of people with an HBV notification in NSW (2010-2017), by HBV DNA testing status**

| **Characteristics** | **Tested within 4 weeks**^§^  **n (%)** | **Tested after 4 weeks**^§^  **n (%)** | **Never tested**^§#^  **n (%)** | **Total**  **n** |
| --- | --- | --- | --- | --- |
| **Total** | **n=5,265 (35%)** | **n=5,103 (33%)** | **n=4,834 (32%)** | **n=15,202** |
| Age at HBV notification, median (IQR) | 38 (30,52) | 35 (28,48) | 37 (29,50) | 37 (29,50) |
| Age at HBV notification |  |  |  |  |
| ≤29 years | 1,450 (33) | 1,629 (37) | 1,329 (30) | 4,408 |
| 30-44 | 1,934 (34) | 1,951 (34) | 1,867 (32) | 5,752 |
| ≥45 years | 1,880 (37) | 1,523 (30) | 1,637 (32) | 5,040 |
| Sex |  |  |  |  |
| Male | 2,937 (35) | 2,640 (32) | 2,760 (33) | 8,337 |
| Female | 2,322 (34) | 2,450 (36) | 2,071 (30) | 6,843 |
| Place of birth^†^ |  |  |  |  |
| Australia | 472 (24) | 499 (26) | 973 (50) | 1,944 |
| Americas, Europe, New Zealand | 227 (27) | 282 (33) | 338 (40) | 847 |
| Africa | 141 (31) | 230 (50) | 85 (19) | 456 |
| Oceania/ East Asia | 2,283 (38) | 2,365 (39) | 1,390 (23) | 6,038 |
| West/ South Asia | 327 (36) | 314 (34) | 276 (30) | 917 |
| Aboriginal and Torres Strait Islander^†^ |  |  |  |  |
| Yes | 74 (16) | 120 (26) | 274 (59) | 468 |
| No | 3,305 (35) | 3,490 (37) | 2,746 (29) | 9,541 |
| History of alcohol-use disorder diagnosis |  |  |  |  |
| Yes | 86 (17) | 128 (25) | 295 (58) | 509 |
| No | 5,179 (35) | 4,975 (34) | 4,539 (31) | 14,693 |
| Local Health District at the time of HBV notification^†^ |  |  |  |  |
| Metropolitan NSW | 2,381 (36) | 2,183 (33) | 1,961 (30) | 6,525 |
| Outer metropolitan NSW | 2,400 (36) | 2,321 (35) | 2,003 (30) | 6,724 |
| Regional/rural NSW | 458 (27) | 550 (32) | 703 (41) | 1,711 |
| History of DC diagnosis |  |  |  |  |
| Yes | 44 (24) | 67 (36) | 74 (40) | 185 |
| No | 5,221 (35) | 5,036 (34) | 4,760 (32) | 15,017 |
| History of HCC diagnosis |  |  |  |  |
| Yes | 57 (40) | 53 (37) | 32 (23) | 142 |
| No | 5,208 (35) | 5,050 (34) | 4,802 (32) | 15,060 |
| Coinfection status |  |  |  |  |
| HBV only | 5,075 (35) | 4,917 (34) | 4,308 (30) | 14,300 |
| HBV/HCV | 159 (20) | 160 (20) | 477 (60) | 796 |
| HBV/HIV | 31 (29) | 26 (25) | 49 (46) | 106 |
| Year of HBV notification |  |  |  |  |
| 2010-2013 | 2,720 (31) | 3,414 (39) | 2,566 (30) | 8,700 |
| 2014-2017 | 2,545 (39) | 1,689 (26) | 2,268 (35) | 6,502 |

DC, decompensated cirrhosis; HBV, hepatitis B virus; HCC, hepatocellular carcinoma; HCV, hepatitis C virus; HIV, human immunodeficiency virus; IQR, interquartile range; NSW, New South Wales

^†^Missing data not shown

^§^Row percentage

^#^Including people who initiated treatment and had no history of prior HBV DNA testing (n=111)

**Table S6: Demographic and liver disease characteristics of people with an HBV notification in NSW (2010-2017), by HBV treatment status**

| **Characteristics** | **Ever Treated**^§^  **n (%)** | **Untreated**^§^  **n (%)** | **Total**  **n** |
| --- | --- | --- | --- |
| **Total** | **n=3,179 (21%)** | **n=12,023 (79%)** | **n=15,202** |
| Age at HBV notification, median (IQR) | 40 (30,53) | 36 (29,49) | 37 (29,50) |
| Age at HBV notification |  |  |  |
| ≤29 years | 836 (19) | 3,572 (81) | 4,408 |
| 30-44 | 1,071 (19) | 4,681 (81) | 5,752 |
| ≥45 years | 1,271 (25) | 3,769 (75) | 5,040 |
| Sex |  |  |  |
| Male | 1,979 (24) | 6,358 (76) | 8,337 |
| Female | 1,198 (18) | 5,645 (82) | 6,843 |
| Place of birth^†^ |  |  |  |
| Australia | 323 (17) | 1,621 (83) | 1,944 |
| Americas, Europe, New Zealand | 158 (19) | 689 (81) | 847 |
| Africa | 95 (21) | 361 (79) | 456 |
| Oceania/ East Asia | 1,546 (26) | 4,492 (74) | 6,038 |
| West/ South Asia | 165 (18) | 752 (82) | 917 |
| Aboriginal and Torres Strait Islander^†^ |  |  |  |
| Yes | 46 (10) | 422 (90) | 468 |
| No | 2,214 (23) | 7,327 (77) | 9,541 |
| History of alcohol-use disorder diagnosis |  |  |  |
| Yes | 79 (16) | 430 (84) | 509 |
| No | 3,100 (21) | 11,593 (79) | 14,693 |
| Local Health District at the time of HBV notification^†^ |  |  |  |
| Metropolitan NSW | 1,466 (22) | 5,059 (78) | 6,525 |
| Outer metropolitan NSW | 1,374 (20) | 5,350 (80) | 6,724 |
| Regional/rural NSW | 302 (18) | 1,409 (82) | 1,711 |
| History of DC diagnosis |  |  |  |
| Yes | 90 (49) | 95 (51) | 185 |
| No | 3,089 (21) | 11,928 (79) | 15,017 |
| History of HCC diagnosis |  |  |  |
| Yes | 101 (71) | 41 (29) | 142 |
| No | 3,078 (20) | 11,982 (80) | 15,060 |
| Coinfection status |  |  |  |
| HBV only | 2,960 (21) | 11,340 (79) | 14,300 |
| HBV/HCV | 131 (16) | 665 (84) | 796 |
| HBV/HIV | 88 (83) | 18 (17) | 106 |
| Year of HBV notification |  |  |  |
| 2010-2013 | 1,985 (23) | 6,715 (77) | 8,700 |
| 2014-2017 | 1,194 (18) | 5,308 (82) | 6,502 |

DC, decompensated cirrhosis; HBV, hepatitis B virus; HCC, hepatocellular carcinoma; HCV, hepatitis C virus; HIV, human immunodeficiency virus; IQR, interquartile range; NSW, New South Wales

^†^Missing data not shown

^§^Row percentage
